# A Skin Cancer Prophylaxis Study in Hairless Mice Using Methylene Blue, Riboflavin, and Methyl Aminolevulinate as Photosensitizing Agents in Photodynamic Therapy

**DOI:** 10.3390/ph14050433

**Published:** 2021-05-05

**Authors:** Hans Christian Wulf, Rami Nabil Al-Chaer, Martin Glud, Peter Alshede Philipsen, Catharina Margrethe Lerche

**Affiliations:** 1 Department of Dermatology, Copenhagen University Hospital, Bispebjerg, DK-2400 Copenhagen, Denmark; h.wulf@regionh.dk (H.C.W.); rami452@icloud.com (R.N.A.-C.); Martin.Glud.01@regionh.dk (M.G.); peter.alshede.philipsen@regionh.dk (P.A.P.); 2 Department of Pharmacy, University of Copenhagen, DK-2100 Copenhagen, Denmark

**Keywords:** PDT, methyl aminolevulinate, methylene blue, riboflavin, photosensitizing agents, ultraviolet radiation, skin tumors, prophylactic treatment, hairless mice

## Abstract

The high incidence of sunlight-induced human skin cancers reveals a need for more effective photosensitizing agents. In this study, we compared the efficacy of prophylactic photodynamic therapy (PDT) when methylene blue (MB), riboflavin (RF), or methyl aminolevulinate (MAL) were used as photosensitizers. All mice in four groups of female C3.Cg/TifBomTac hairless immunocompetent mice (*N* = 100) were irradiated with three standard erythema doses of solar-simulated ultraviolet radiation (UVR) thrice weekly. Three groups received 2 × 2 prophylactic PDT treatments (days 45 + 52 and 90 + 97). The PDT treatments consisted of topical administration of 16% MAL, 20% MB, or 20% RF, and subsequent illumination that matched the photosensitizers’ absorption spectra. Control mice received no PDT. We recorded when the first, second, and third skin tumors developed. The pattern of tumor development after MB-PDT or RF-PDT was similar to that observed in irradiated control mice (*p* > 0.05). However, the median times until the first, second, and third skin tumors developed in mice given MAL-PDT were significantly delayed, compared with control mice (256, 265, and 272 vs. 215, 222, and 230 days, respectively; *p* < 0.001). Only MAL-PDT was an effective prophylactic treatment against UVR-induced skin tumors in hairless mice.

## 1. Introduction

Photodynamic therapy (PDT) was discovered more than 100 years ago and has since become well established [1]. It is effective in the treatment of various human skin cancers and precancerous lesions [1,2,3]. PDT involves a photosensitizer that is activated by ultraviolet radiation (UVR) and/or visible light to initially form an excited singlet state, followed by transition to a long-lived excited triplet state [3]. This triplet state can undergo photochemical reactions in the presence of oxygen to form reactive oxygen species that can destroy skin cells [3]. The dual specificity of PDT relies on the accumulation of the photosensitizing agent in diseased tissue, and on localized light delivery [2]. The subcellular localization of the photosensitizing agent (e.g., in the mitochondria, lysosomes, endoplasmic reticulum, or plasma membrane) probably plays a major role in the mechanism of cell death that predominates, but other factors, such as the concentration of the photosensitizer, absorption coefficient of the photosensitizer, light dose (J/cm^2^), and duration of light exposure, may also be important [1]. The molecular mechanism in PDT prophylaxis is the same as in PDT treatment, but targeting subclinical dysplatic cells instead of regular actinic keratosis or squamous cell carcinoma.

Chemical properties, such as lipophilicity, molecule size, and viscosity, affect the tissue biodistribution of photosensitizing agents [1]. Anticancer photosensitizing agents tend to be lipophilic, with little or no overall charge [1]. Many synthetic and natural non-toxic dyes have been investigated, with a view to implementing these in PDT. Methyl aminolevulinate (MAL) is a commonly used photosensitizer in PDT. However, research is ongoing to identify better alternatives. PDT with MAL is a cosmetically attractive alternative to conventional destructive treatments, including cryotherapy or surgical removal of skin cancers, such as precancerous actinic keratoses [4]. However, clinical use of PDT with MAL has been limited due to adverse side effects, the frequent recurrence of thick skin lesions, and a relapse rate of between 14 and 33% [5,6,7,8,9,10,11]. The treatment time is crucial in the clinic, and MAL needs to be converted to protoporphyrin IX (PpIX) in the cell before it is active. Alternative compounds could act as a photosensitizer directly, without cellular conversion.

The photosensitizing agents methylene blue (MB) and riboflavin (RF), also known as vitamin B2, are currently being assessed as potential treatments for UVR-induced skin cancer [12,13]. An RF derivate was tested for its antimicrobial and anticancer properties, and showed efficacy against malignant melanoma in an in vivo mouse model, and fewer side effects than treatments currently in use [3]. MB is a phenothiazinium dye that has been studied frequently for its antimicrobial applications, and occasionally for its anticancer activity [1].

In this study, we investigated the prophylactic effect of RF-PDT and MB-PDT, compared with MAL-PDT, in delaying the development of UVR-induced skin tumors in hairless mice.

## 2. Results

### 2.1. Efficacy of Prophylactic PDT

**Figure 1:**
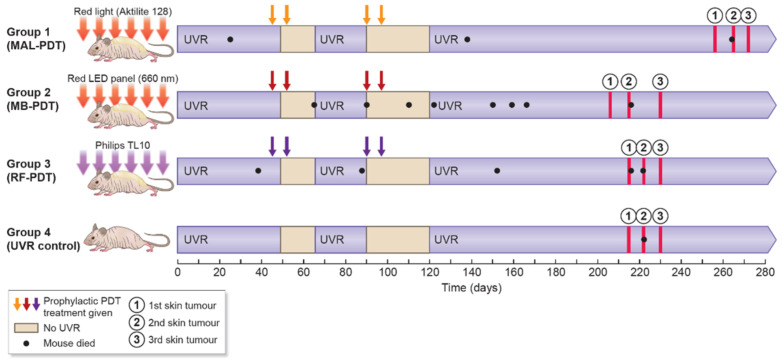
Study overview. Abbreviations: MAL, methyl aminolevulinate; MB, methylene blue; RF, riboflavin; PDT, photodynamic therapy; LED, light-emitting diode; UVR, ultraviolet radiation.

**Figure 2:**
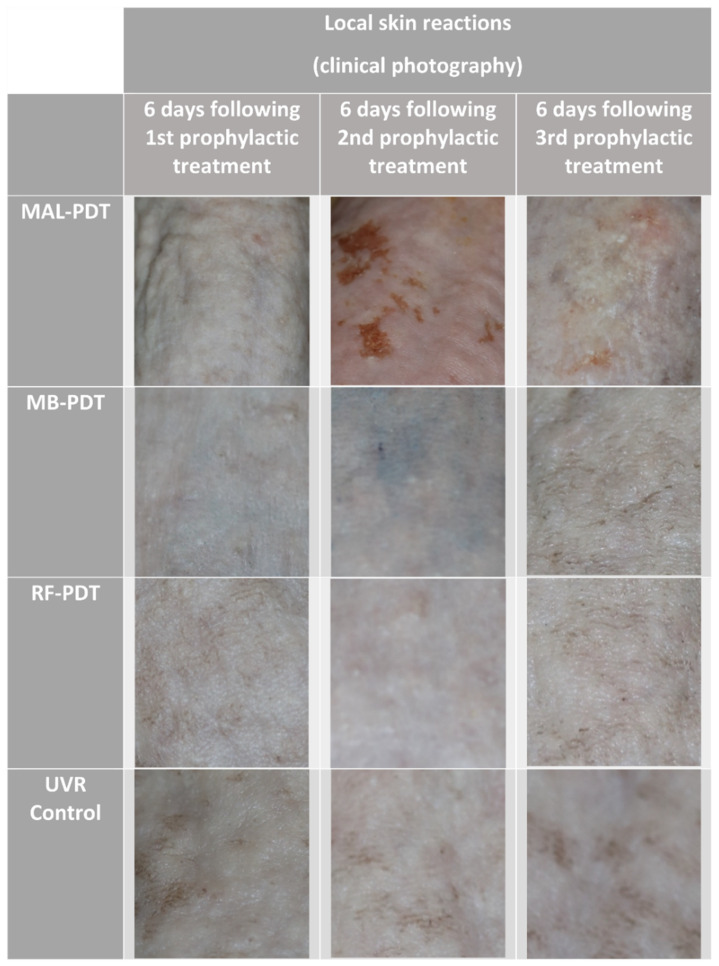
Clinical reactions after the prophylactic treatments. Abbreviations: MAL, methyl aminolevulinate; MB, methylene blue; RF, riboflavin; PDT, photodynamic therapy; UVR, ultraviolet radiation.

Four groups of mice were treated as shown in Figure 1. Mice treated with MB-PDT and RF-PDT showed no signs of severe discomfort during the illuminations, and no local skin reactions were observed over the following days (Figure 2). In contrast, mice treated with MAL-PDT did show discomfort during the illuminations, and erythema of the skin was observed on the day after treatment. Small wounds developed after the second MAL-PDT (day 52) and fourth MAL-PDT (day 97) treatments (Figure 2). Therefore, UVR treatments for all groups were discontinued between days 45 and 66, and between days 90 and 120.

**Figure 3:**
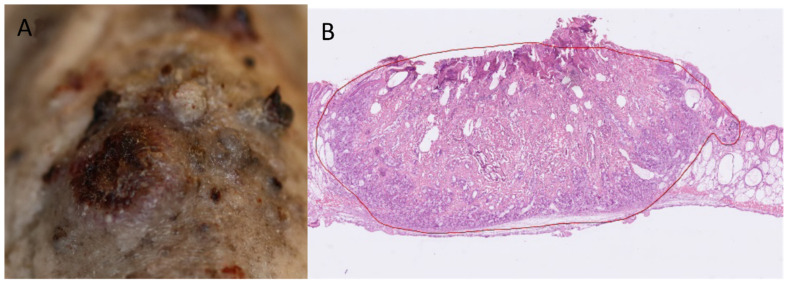
(**A**) Clinical photo of a representative tumor. (**B**) Tumor histology showing squamous cell carcinoma (stained with hematoxylin and eosin).

**Figure 4:**
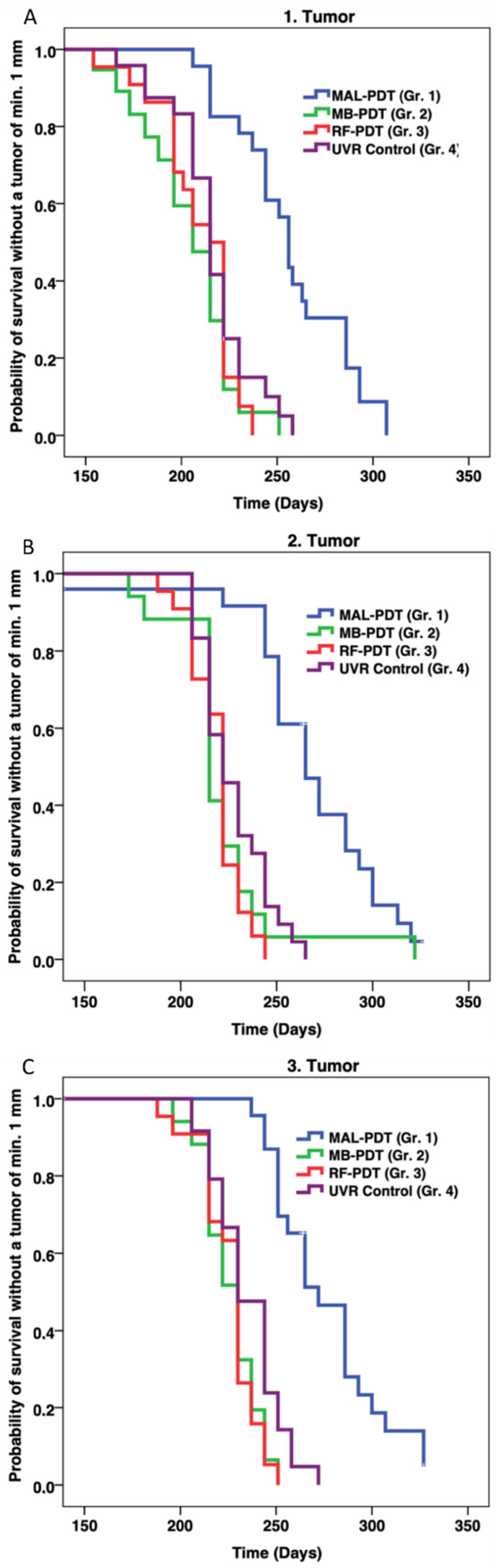
Kaplan–Meier plots showing the probability of survival without a first (**A**), second (**B**), and third (**C**) tumor (minimum diameter = 1 mm) as a function of time. Abbreviations: MAL, methyl aminolevulinate; MB, methylene blue; RBF, riboflavin; Gr., group.

The weekly UVR continued until each mouse developed three 4 mm tumors or one 12 mm tumor. Histological analyses of two random tumors from different mice in each of the four groups confirmed a diagnosis of squamous cell carcinoma in all cases (Figure 3). There were no statistically significant differences in when the first tumors developed in mice from groups MB-PDT and RF-PDT, compared with mice from the UVR control group: (206 vs. 215 days, *p* = 0.160) and (215 vs. 215 days, *p* = 0.394), respectively (Figure 4 and Table 1). There was no significant difference in when the first tumor developed between mice in the MB-PDT and RF-PDT groups (206 vs. 215 days, *p* = 0.491). However, mice treated with MAL-PDT exhibited a significant delay before the first tumor developed, compared with the UVR control group (256 vs. 215 days, *p* = 0.000004; Figure 4 and Table 1). Statistically significant differences in when the first tumors developed were also found when mice from the MB-PDT and RF-PDT groups were compared with those from the MAL-PDT group: (206 vs. 256, *p* = 1.6 × 10^−7^) and (215 vs. 256, *p* = 5.3 × 10^−7^), respectively.

The same pattern was observed for the development of the second and third tumors. Mice in the MB-PDT, RF-PDT, and UVR control groups developed a second and third tumor at approximately the same times (*p* > 0.061) (Table 1 and Figure 4). Mice in the MAL-PDT exhibited a significant delay before the second and third tumors developed, compared with the other groups (*p* ≤ 0.000199).

No weight differences were observed among the groups (*p* > 0.05, results not shown). However, significantly more mice died before developing a first tumor in the MB-PDT group than in the UVR control group (*p* = 0.048). Three mice in the MAL-PDT group, seven mice in the MB-PDT group, five mice in the RF-PDT group, and one mouse in the UVR control group died before reaching the endpoint (Figure 1).

### 2.2. Pigmentation

**Figure 5:**
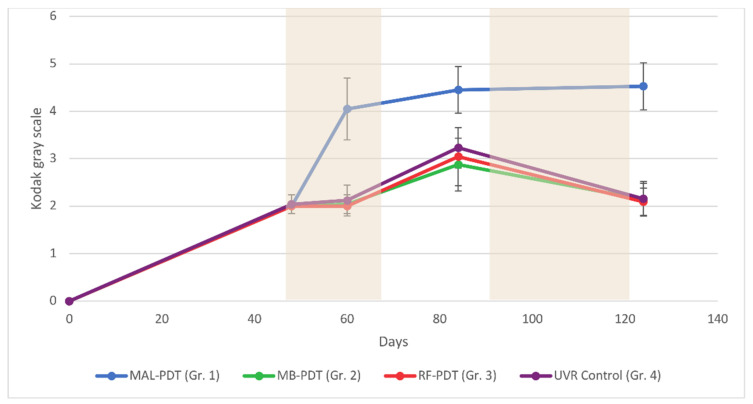
Pigmentation for all groups of mice measured on a Kodak gray scale (arbitrary units, au) and analyzed using one-way analysis of variance (ANOVA). The shaded areas indicate periods with no exposure to ultraviolet irradiation. Abbreviations: MAL, methyl aminolevulinate; MB, methylene blue; RF, riboflavin; Gr., group.

All UVR-irradiated mice developed pigmented skin (Figure 2 and Figure 5). At days 0 and 48, there were no differences in pigmentation among the four groups (*p* > 0.05). However, after the first two PDT treatments, a significant increase in skin pigmentation was observed in mice treated with MAL-PDT (day 124: mean, 4.5 (range, 4–5)). Skin pigmentation in the MAL-PDT group was significantly greater than in the other groups at days 60 (*p* < 0.0001), 84 (*p* < 0.0001), and 124 (*p* < 0.0001).

There were no significant differences in skin pigmentation among the MB-PDT, RF-PDT, and UVR control groups at any time (*p* > 0.05). Similar decreases in skin pigmentation were observed in the MB-PDT, RF-PDT, and UVR control groups when UVR exposure was discontinued between days 90 and 120. This decrease in pigmentation was not observed in the MAL-PDT group.

## 3. Discussion

The present study investigated whether prophylactic PDT, with different photosensitizing agents being applied to the skin of hairless mice, could delay UVR-induced photocarcinogenesis. We used C3.Cg/TifBomTac hairless mice, which develop pigmentation after treatment with UVR, and therefore mimic the human response to UVR better than SKH-1 mice. As anticipated, we found that chronic UVR exposure strongly induced skin cancer in the murine model.

**Figure 6:**
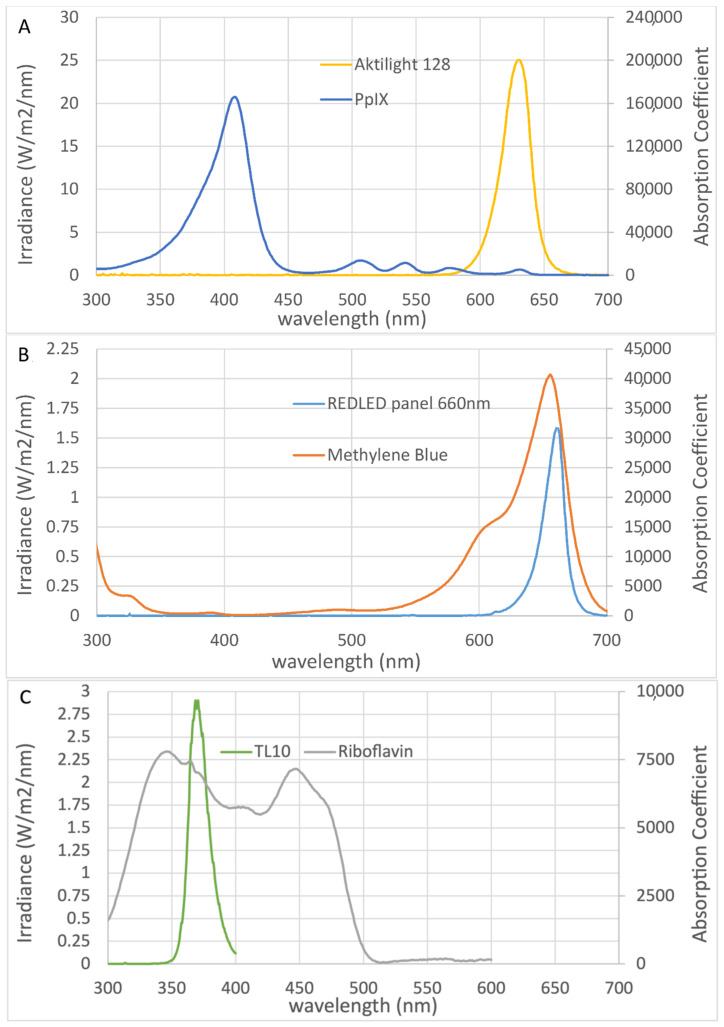
Absorption and irradiance spectra of the following photosensitizers and corresponding light sources: (**A**) Protoporphyrin IX (PpIX), (**B**) methylene blue, and (**C**) riboflavin. The absorption spectra shown were created using PhotochemCADTM software [26,27].

Solar UVR consists predominantly of ultraviolet-A (UVA; >90%; 400–315 nm) and ultraviolet-B (UVB; <10%; 315–280 nm) [14]. UVR has direct effects on genomes and stimulates inflammation [14,15], and UVB is more carcinogenic than UVA [16]. In this murine model, we found that prophylactic PDT treatment with MB (MB-PDT) or RF (RF-PDT) did not delay the development of the first, second, and third tumors, compared with mice that only received UVR (UVR controls). Neither treatment showed efficacy in the prophylaxis of UVR-induced skin tumors in hairless mice. As described in the introduction, many factors are important when considering a photosensitizer for PDT. Previous research has demonstrated that MB and RF are absorbed by the skin or skin models after topical application, although neither is as lipophilic as MAL [17,18], but there are also studies showing no uptake of MB in skin without pretreatment [19,20], which could be due to the positive charge of the molecule. Ideally, light sources should exhibit maximal output at wavelengths matching the photosensitizers’ maximal absorption. The wavelength of light determines the depth to which it penetrates the skin. Figure 6 shows the irradiance spectra of our light sources and the absorption spectra of the photosensitizers that were used. MAL is converted to PpIX in the cell. Blue light, red light, and daylight can all be used with MAL for PDT. Figure 6A shows that a greater dose of red light is required than blue for treatment with MAL because PpIX absorbs light more strongly in the blue region of the spectrum. However, red light penetrates the skin to a greater depth. Moreover, the absorption coefficient of the photosensitizer is an important factor, and it can be seen from Figure 6 that the absorption factors for PpIX and RF are much lower than for MB at the irradiation wavelengths, which cannot explain the poor outcome of the MB and RF treatments. Figure 6C indicates that using a light source matching the 440 nm rather than the 375 nm peak may have increased skin penetration and improved RF-PDT. Using another light source for RF-PDT with a broader absorption spectrum than TL10 could also have improved the outcome.

More mice died in the MB-PDT treated group than in the UVR control group. The reason for this significant increase in mortality is unclear, but it may be due to mice ingesting some of the photosensitizing agent applied to their skin. Although MB is poorly absorbed in the gastrointestinal tract, it may generate potentially fatal levels of serotonin at doses >5 mg/kg, resulting in anaphylactic shock [21]. We applied 720 mg/kg MB to the skin of each mouse at 2 × 2 prophylactic treatments.

The results observed in the MAL-PDT group of mice were consistent with those reported by previous studies, and confirm that tumor development can be delayed in this mouse model [2,22]. We assume that the molecular mechanism behind the effect is the same as for PDT treatment with light-mediated excitation of photosensitizer-loaded tumor cells resulting in the production of reactive oxygen species within these cells, leading to cell death. However, since the treatment is given before tumor development (day 45 and 90), the target is subclinical dysplastic cells, instead of regular actinic keratosis or squamous cell carcinoma.

The mice in the MAL-PDT group developed the most intense pigmentation, measured using the Kodak gray scale, probably due to postinflammatory hyperpigmentation. This indicates that MAL-PDT produces an intense PDT effect, as reported by previous studies [23]. The intense pigmentation may partly explain the delay in tumor development, although tumors do appear if UVR treatment is prolonged, despite the high levels of pigmentation [24,25].

Silva et al. demonstrated that application of a 1% MB solution, followed by irradiation with a diode laser for 15 min at 74 mW/cm^2^ (total dose = 100 J/cm^2^) could reduce tumor size. However, this treatment also reduced epithelial thickness, and keratinization occurred on the surrounding healthy skin tissue [13]. On the other hand, Khaydukov et al. demonstrated that using RF as a photosensitizer generated reactive oxygen species under UVR/blue light irradiation and killed cancer cells [12]. This phototoxicity was demonstrated using the human breast adenocarcinoma cell line SK-BR-3 [12]. We investigated the efficacy of different photosensitizing agents in prophylactic PDT treatments against UVR-induced precancerous skin changes. However, our findings are not consistent with the studies described above. Neither MB-PDT nor RF-PDT delayed the development of precancerous skin changes. It could be ideal to develop photosensitizers that are directly active without a conversion to PpIX to save time in the clinic, but, with suboptimal photosensitizer properties, MB and RF are not the best candidates as potential photosensitizers for topical use on skin cancers and precursors.

In conclusion, we found that, whereas prophylactic treatment with MB-PDT or RF-PDT was ineffective, treatment with MAL-PDT did delay the development of UVR-induced skin tumors in hairless mice.

## 4. Materials and Methods

### 4.1. Animals

Four groups of 25 female C3.Cg/TifBomTac immunocompetent mice (*N* = 100) were included in the study. They were 12–16 weeks old at the beginning of our experiment. These mice were used because they are hairless and can develop UVR-induced pigmentation, mimicking the response of human skin to UVR. All mice were tattooed with consecutive numbers on the abdomen, and each group of mice was housed in a separate box where there was free access to drinking water and standard laboratory food. The mice were kept at 23–24 °C under a 12-h light/dark cycle.

### 4.2. Study Design

The mice were randomly divided into four groups of 25 mice. The first four tumors with a diameter of at least 1 mm were mapped separately for each mouse and followed until three of these reached a diameter of 4 mm. Mice were examined for tumors every week. The “time to the first tumor” was the number of days it took for the first 1 mm diameter tumor that later grew to 4 mm to appear. As secondary endpoints, we also recorded when the second and third tumors appeared using the same principle. Mice were euthanized when they had developed three 4 mm tumors, one 12 mm tumor, or after 365 days. After the mice were sacrificed, one tumor was taken from two randomly selected mice in each group, mounted in Tissue-Tek optimal cutting temperature compound (Sakura Finetek Europe B.V., Alphen aan den Rijn, The Netherlands), and frozen. Biopsies were sliced vertically into 10 mm thick sections for hematoxylin and eosin staining and evaluated by a Mohs surgeon. Weight and skin pigmentation were measured once every month. Pigmentation was quantified on a 20-point categorical scale of arbitrary units (au) using the Kodak gray scale.

### 4.3. UVR Exposure

Mice were irradiated with three standard erythema doses (SEDs) three times a week from the beginning of the study. The UVR source consisted of one UV6 tube (Waldmann, Wheeling, IL, USA) placed between five Bellarium-S SA-1-12 tubes (Wolff System, Atlanta, GA, USA). The animals were irradiated from above, in their boxes, through wire lids. Distances were adjusted every month to maintain the required doses. UVR doses were measured in SEDs using a spectroradiometer (Solatell Sola-Hazard 4D Controls Ltd., Cornwall, UK) [28]. For comparison, a Danish summer midday sun typically generates a UVR dose of 3 SEDs in 30 min [29].

### 4.4. Photosensitizers

Prophylactic PDT treatments were given at days 45 and 52, and again at days 90 and 97. The prophylactic treatment regimen was based on previous experience from a similar study in mice [23]. The skin was not prepared prior to the prophylactic treatments, and 100 µL of topical photosensitizer was applied to the dorsal skin of each mouse (from front legs to tail). The spectra of the illumination sources were recorded using a spectroradiometer (Jaz, Ocean Optics, FL, USA).

The photosensitizer used on group 1 mice was 16% MAL cream (Metvix^®^; Galderma, Lausanne, Switzerland). For MAL-PDT treatment, the animals were kept in a dark room for 3 h after the cream was applied, and any remaining cream was wiped off. The standard red light illumination (38 J/cm^2^) was administered from above for approximately 9 min using a light-emitting diode (LED) lamp (Aktilite 128; Photocure ASA, Oslo, Norway; Figure 6A). The largest absorption peak for MAL is at 410 nm, with several smaller peaks between 500 and 635 nm [4].

The photosensitizer used on group 2 mice was 20% (*w*/*w*) MB (Sigma-Aldrich, St Louis, MO, USA) in a cream base (Unguentum M; Almirall, Reinbek, Germany). The illuminations commenced 0.5 h after the cream was applied. The light source was a red LED panel (A LED, Varde, Denmark), with peak intensity at 660 nm. MB absorbs light most strongly in the red spectral region, with a peak at 660 nm (Figure 6B). A light dose of 37 J/cm^2^ was administered for 3 h.

The photosensitizer used on group 3 mice was 20% (*w*/*w*) RF (Sigma-Aldrich, St Louis, MO, USA) in a cream base (Unguentum M; Almirall, Reinbek, Germany). The animals were kept in a dark room for 1 h after the cream was applied. The light source was a panel of Philips TL10 tubes (Figure 6C). RF has absorption peaks in the UVA (360 nm) and blue (440 nm) spectral regions. A light dose of 37 J/cm^2^ was administered for 2 h.

### 4.5. Statistics

We used both parametric and nonparametric statistical analyses, and reported descriptive data as medians with percentiles. The time to onset of the first, second, and third tumors was visualized in Kaplan–Meier plots. Groups were compared using log-rank tests (Mantel–Cox). Weight and skin pigmentation were analyzed by one-way analysis of variance (ANOVA), followed by Dunn’s multiple comparison test for groupwise post hoc comparisons. Mortality before the first tumor was assessed among groups using chi-square tests. *P*-values less than 0.05 were considered significant. All analyses were performed using IBM SPSS 25 software for Windows (SPSS Inc., Chicago, IL, USA).

## 5. Conclusions

In conclusion, prophylactic treatment with MAL-PDT delayed the development of UVR-induced skin tumors in hairless mice but MB-PDT and RF-PDT was ineffective.

## Figures and Tables

**Table 1:** Treatment schedule and results. Median number of days to onset of the first, second, and third tumors in 50% of the mice in each of the four groups. Interquartile ranges (25th and 75th percentiles) are shown.

**Group No.**	1	2	3	4
**No. of Mice (*n*)**	25	25	25	25
**No. of Mice (*n*) Dy-ing Before End-Point**	3	7	5	1
**Treatment**	MAL-PDT p-value *	MB-PDT p-value *	RF-PDT p-value *	UVR Control
**Median Days to 1st Tumor**	256 (237–286) 0.000004	206 (188–222) 0.160	215 (196–222) 0.394	215 (206–222)
**Median Days to 2nd Tumor**	265 (251–293) $3.1\cdot 10^{-7}$	215 (215–230) 0.526	222 (206–222) 0.111	222 (215–244)
**Median Days to 3rd tumor**	272 (251–293) $3.6\times 10^{-7}$	230 (215–237) 0.061	230 (215–237) 0.060	230 (222–244)

* *p*-value for the group in question is compared with the control group. Abbreviations: MAL, methyl aminolevulinate; MB, methylene blue; RF, riboflavin; PDT, photodynamic therapy; UVR, ultraviolet radiation.

## Data Availability

The data presented in this study are available on request from the corresponding author.
